# Asciminib monotherapy in patients with chronic-phase chronic myeloid leukemia with the T315I mutation after ≥1 prior tyrosine kinase inhibitor: 2-year follow-up results

**DOI:** 10.1038/s41375-024-02278-8

**Published:** 2024-05-16

**Authors:** Jorge E. Cortes, Koji Sasaki, Dong-Wook Kim, Timothy P. Hughes, Gabriel Etienne, Michael J. Mauro, Andreas Hochhaus, Fabian Lang, Michael C. Heinrich, Massimo Breccia, Michael Deininger, Yeow Tee Goh, Jeroen J.W.M. Janssen, Moshe Talpaz, Valle Gomez Garcia de Soria, Philipp le Coutre, Daniel J. DeAngelo, Andrea Damon, Silvia Cacciatore, Fotis Polydoros, Nithya Agrawal, Delphine Rea

**Affiliations:** 1 Georgia Cancer Center at Augusta University, Augusta, GA USA; 2 Department of Leukemia, The University of Texas MD Anderson Cancer Center, Houston, TX USA; 3 Uijeongbu Eulji Medical Center, Geumo-dong, Uijeongbu-si, South Korea; 4 South Australian Health and Medical Research Institute and University of Adelaide, Adelaide, SA Australia; 5 Department of Hematology, Institut Bergonié, Bordeaux, France; 6 Myeloproliferative Neoplasms Program, Memorial Sloan Kettering Cancer Center, New York, NY USA; 7 Hematology/Oncology, Universitätsklinikum Jena, Jena, Germany; 8 Department of Medicine, Hematology and Oncology, Goethe University Hospital, Frankfurt, Germany; 9 Portland VA Health Care System and OHSU Department of Medicine, Division of Hematology and Oncology, Knight Cancer Institute, Portland, OR USA; 10 Department of Translational and Precision Medicine-Az., Policlinico Umberto I-Sapienza University, Rome, Italy; 11 Versiti Blood Research Institute, Milwaukee, WI USA; 12 Department of Haematology, Singapore General Hospital, Bukit Merah, Singapore; 13 Radboud University Medical Center, Nijmegen, The Netherlands; 14 Division of Hematology-Oncology, University of Michigan Rogel Cancer Center, Ann Arbor, MI USA; 15 Hospital Universitario La Princesa, Madrid, Spain; 16 Department of Oncology and Hematology, Charité - Universitätsmedizin Berlin, Berlin, Germany; 17 Dana-Farber Cancer Institute, Boston, MA USA; 18 Novartis Pharmaceuticals Corporation, East Hanover, NJ USA; 19 Novartis Pharma AG, Basel, Switzerland; 20 Department of Hématologie, Hôpital Saint-Louis, Paris, France

**Keywords:** Chronic myeloid leukaemia, Molecularly targeted therapy

## Abstract

Asciminib targets the *BCR::ABL1* myristoyl pocket, maintaining activity against *BCR::ABL1*^T315I^, which is resistant to most approved adenosine triphosphate–competitive tyrosine kinase inhibitors. We report updated phase I results (NCT02081378) assessing safety/tolerability and antileukemic activity of asciminib monotherapy 200 mg twice daily in 48 heavily pretreated patients with T315I-mutated chronic-phase chronic myeloid leukemia (CML-CP; data cutoff: January 6, 2021). With 2 years’ median exposure, 56.3% of patients continued receiving asciminib. Overall, 62.2% of evaluable patients achieved *BCR::ABL1* ≤1% on the International Scale (IS); 47.6% and 81.3% of ponatinib-pretreated and -naive patients, respectively, achieved *BCR::ABL1*^IS^ ≤1%. Of 45 evaluable patients, 48.9% achieved a major molecular response (MMR, *BCR::ABL1*^IS^ ≤0.1%), including 34.6% and 68.4% of ponatinib-pretreated and -naive patients, respectively. MMR was maintained until data cutoff in 19 of 22 patients who achieved it. The most common grade ≥3 adverse events (AEs) included increased lipase level (18.8%) and thrombocytopenia (14.6%). Five (10.4%) patients experienced AEs leading to discontinuation, including 2 who discontinued asciminib and died due to COVID-19; these were the only deaths reported. These results show asciminib’s effectiveness, including in almost 50% of ponatinib pretreated patients, and confirm its risk-benefit profile, supporting its use as a treatment option for T315I-mutated CML-CP.

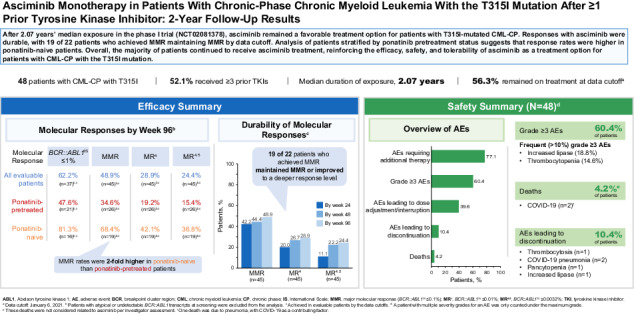

## Introduction

*BCR::ABL1* tyrosine kinase inhibitors (TKIs) have improved survival for patients with chronic myeloid leukemia (CML) [1, 2]. However, many patients experience resistance or intolerance through successive adenosine triphosphate (ATP)–competitive TKI therapies, resulting in decreased probability of survival [2–6]. A common mechanism of resistance is emerging *BCR::ABL1* mutations [2, 7]. The T315I mutation is among the most frequently identified *BCR::ABL1* mutations, occurring in 2% to 16% of patients with imatinib- or second-generation TKI-resistant CML and increasing in frequency with subsequent lines of therapy [8, 9]. The T315I mutation confers resistance to all currently approved ATP-competitive TKIs except ponatinib and olverembatinib (approved in China for TKI-resistant CML in chronic phase [CP] or accelerated phase [AP] with the T315I mutation), limiting treatment options for affected patients [1, 2, 10–12].

Patients with the T315I mutation may have adverse outcomes, including decreased overall survival (OS) and progression-free survival [8, 9, 13, 14]. Ponatinib has demonstrated activity in patients, although it may be associated with safety concerns [1, 13, 15–18]. In the OPTIC trial, which assessed ponatinib starting doses of 45, 30, and 15 mg QD, 51.6%, 35.5%%, and 25.3%, respectively, of patients with T315I-mutated CML-CP achieved *BCR::ABL1* on the International Scale (IS) ≤1% by 12 months (predictive of long-term survival) [1, 16, 19, 20]. Safety concerns associated with ponatinib include the risk of cardiovascular (CV) adverse events (AEs), including arterial occlusive events (AOEs), in 14% to 31% of patients with CML-CP, although rates can be reduced by approximately 60% using response-based dose-reduction strategies [1, 13, 18, 21].

Asciminib is the first approved *BCR::ABL1* inhibitor that works by specifically targeting the ABL myristoyl pocket, inhibiting *BCR::ABL1* kinase activity by locking it in an inactive conformation via allosteric binding [12, 22–25]. Asciminib has high specificity and selectivity for the ABL kinase family with limited off-target activity [12, 23]. By targeting the myristoyl-binding pocket, asciminib maintains activity against *BCR::ABL1* kinase domain mutations, including T315I, that confer resistance to ATP-competitive TKIs [12, 22, 23, 26].

A previous analysis of the phase I X2101 trial (NCT02081378) in heavily pretreated patients with Ph+ CML-CP/AP, including T315I-mutated CML-CP/AP, first demonstrated the safety of asciminib monotherapy QD or twice daily (BID) at 10 to 200 mg [26]. After a median follow-up of approximately 14 months, asciminib demonstrated a favorable safety and tolerability profile, with 11% and 6% of patients with CML-CP with or without the T315I mutation, respectively, discontinuing therapy due to AEs [26]. The maximum tolerated dose of asciminib was not reached [26]. Preclinical observations suggested that a 4- to 13-fold higher asciminib concentration was required for adequate inhibition of *BCR::ABL1*^T315I^ compared with non-mutated *BCR::ABL1*, and in X2101 the majority of patients with T315I-mutated CML-CP who achieved responses had received doses of ≥150 mg BID [12, 23, 26, 27]. Use of the highest tested dose of asciminib, 200 mg BID, was justified by overall major molecular response (MMR; *BCR::ABL1*^IS^ ≤0.1%) rates predicted by pharmacokinetic/pharmacodynamic analysis in patients with T315I-mutated CML-CP and may improve the probability of response by maximizing the predicted proportion of patients with asciminib exposure above the preclinical 90% maximal effective concentration [28]. Results from this trial supported full approval of asciminib 200 mg BID for patients with T315I-mutated CML-CP in the US with subsequent approvals worldwide [29–31].

We report efficacy and safety data in 48 patients with T315I-mutated CML-CP receiving asciminib 200 mg BID monotherapy with a median duration of exposure of 2 years in X2101; separate analyses of patients with CML-CP receiving asciminib 150 or 160 mg and patients with CML-AP receiving asciminib 200 mg BID are also included.

## Methods

### Study oversight

The study was designed by the sponsor (Novartis Pharmaceuticals) in collaboration with study investigators. The sponsor collected and analyzed data in conjunction with the authors. All authors contributed to the development and writing of the manuscript and vouch for the accuracy and completeness of the data and the fidelity of the study to the protocol.

### Study design

This phase I, first-in-human, dose-finding study was described previously [25, 26]. The current analysis focused on 48 patients with CML-CP with a confirmed T315I mutation who received a starting dose of asciminib monotherapy 200 mg BID in the dose-escalation or -expansion phases of this study arm. *BCR::ABL1* mutational analyses were performed centrally by ICON (Portland, OR, USA) using Sanger sequencing. Patients were ≥18 years old with cytogenetically confirmed Ph+ CML-CP with the T315I mutation who were resistant to or intolerant of ≥1 prior TKI (per 2009 European LeukemiaNet recommendations) [32]. Efficacy and safety data for patients with T315I-mutated CML-CP who received asciminib 150 mg BID (n = 5) and 160 mg BID (n = 6) and efficacy data for patients with T315I-mutated CML-AP who received asciminib 200 mg BID (n = 4) are reported. The primary objective was to determine the maximum tolerated dose and/or recommended dose for expansion of asciminib monotherapy. Secondary objectives included assessing the safety and tolerability, pharmacokinetics, and preliminary antileukemic activity of asciminib.

### Study assessments

Coding and grading of AEs, assessment of molecular response, and *BCR::ABL1* mutational analysis were described previously [25].

### Statistical analyses

Assessment of molecular response rates by time point was defined previously [25]. The analyses herein are based on data collected by the January 6, 2021, cutoff, when all patients had completed their week 60 follow-up visit or discontinued earlier and represent cumulative rates unless otherwise specified.

## Results

### Patients

This analysis included all 48 patients, enrolled from October 2016 to October 2019, with T315I-mutated CML-CP receiving asciminib monotherapy 200 mg BID (Supplemental Fig. S1). Two patients had additional mutations at baseline (E255K [n = 1], E355G [n = 1]). At data cutoff, more than half of patients (27 [56.3%]) remained on treatment (Table 1). With 2.07 years (range, 0.04–4.12) median follow-up and median duration of exposure, 21 (43.8%) patients discontinued treatment, primarily due to AEs (8.3%) and physician decision (22.9%), including loss of response (n = 3), loss/lack of response followed by stem cell transplant (n = 3), lack of efficacy (n = 2), to receive stem cell transplant (n = 2), and transfer to investigator-initiated trial (n = 1). Thirty-six (75.0%) and 27 (56.3%) patients received treatment for ≥48 and ≥96 weeks, respectively; most (77.8% and 74.1%, respectively) were receiving 200 mg BID at those cutoffs (Supplemental Table S1). Fifteen (31.3%) patients received treatment for ≥144 weeks. Two (4.2%) patients died on study; one died on treatment (within 30 days of last study drug dose) due to COVID-19 pneumonia, and the other died >30 days post study discontinuation due to pneumonia with COVID-19 as the contributing reason.

**Table 1:** Patient disposition.

Disposition reason, n (%)	Ponatinib pretreated n = 29	Ponatinib naive n = 19	All patients N = 48
Patients treated			
Treatment ongoing^a^	15 (51.7)	12 (63.2)	27 (56.3)
End of treatment	14 (48.3)	7 (36.8)	21 (43.8)
Primary reason for end of treatment			
Physician decision^b^	8 (27.6)	3 (15.8)	11 (22.9)
Adverse event	2 (6.9)	2 (10.5)	4 (8.3)
Patient/guardian decision	0	0	0
Progressive disease	2 (6.9)^c^	0	2 (4.2)^c^
Death	0	2 (10.5)	2 (4.2)
Technical problems	1 (3.4)^d^	0	1 (2.1)^d^
Protocol deviation	1 (3.4)^e^	0	1 (2.1)^e^

^a^Patients ongoing at the time of the cutoff (January 6, 2021).

^b^Reasons for physician decision included loss of response (n = 3), loss or lack of response with transfer to stem cell transplant (n = 3), lack of efficacy (n = 2), to receive stem cell transplant (n = 2), and transfer to investigator-initiated trial (n = 1).

^c^Patients with disease progression to accelerated phase.

^d^Patient travel to trial site restricted due to COVID-19. Moved to a managed access program.

^e^Patient required prohibited medication (hydroxyurea). Continued treatment under a managed access program.

Most patients were heavily pretreated, with 15 (31.3%), 17 (35.4%), and 8 (16.7%) having received 2, 3, and ≥4 prior TKIs, respectively (Table 2). No patients previously received olverembatinib. Twenty-nine (60.4%) patients previously received ponatinib (Supplementary Table S2); these patients received a median of 3 (range, 1–5) prior TKIs. Eleven of 26 patients with available prior ponatinib dosing data received an initial ponatinib dose of 45 mg. Twenty-six of 29 patients received ponatinib as the last TKI before study entry. Patients discontinued ponatinib due to intolerance (n = 9), resistance (n = 14), enrollment in X2101 (n = 4), or completion of prescribing regimen (n = 2). Baseline *BCR::ABL1*^IS^ levels of 26 evaluable patients separated by reason for ponatinib discontinuation are shown in Supplementary Table S3.

**Table 2:** Patient demographics and baseline clinical characteristics.

Demographic variable	All patients N = 48	Evaluable patients N = 45
Age, median (range), years	56.5 (26–86)	54.0 (26–86)
Age category, n (%)		
18 to <65 years	32 (66.7)	31 (68.9)
≥65 years	16 (33.3)	14 (31.1)
≥75 years	4 (8.3)	4 (8.9)
Sex, n (%)		
Male	37 (77.1)	36 (80.0)
Female	11 (22.9)	9 (20.0)
Race, n (%)		
White	23 (47.9)	21 (46.7)
Asian	12 (25.0)	12 (26.7)
Unknown	6 (12.5)	5 (11.1)
Other	6 (12.5)	6 (13.3)
Black or African American	1 (2.1)	1 (2.2)
Ethnicity, n (%)		
East Asian	10 (20.8)	10 (22.2)
Other	16 (33.3)	14 (31.1)
Not reported	14 (29.2)	13 (28.9)
Unknown	3 (6.3)	3 (6.7)
Southeast Asian	2 (4.2)	2 (4.4)
Hispanic or Latino	3 (6.3)	3 (6.7)
ECOG performance status, n (%)		
0	36 (75.0)	33 (73.3)
1	12 (25.0)	12 (26.7)
No. of prior TKIs		
1^a^	8 (16.7)	8 (17.8)
2	15 (31.3)	14 (31.1)
3	17 (35.4)	16 (35.6)
≥4	8 (16.7)	7 (15.6)
Individual prior TKIs		
Bosutinib	3 (6.3)	2 (4.4)
Dasatinib	33 (68.8)	33 (73.3)
Imatinib	27 (56.3)	25 (55.6)
Nilotinib	26 (54.2)	23 (51.1)
Ponatinib	29 (60.4)	26 (57.8)
Radotinib	4 (8.3)	4 (8.9)
Reason for discontinuation of last dose of ponatinib		
Intolerance	9 (31.0)	7 (26.9)
Resistance	14 (48.3)	13 (50.0)
Other	6 (20.7)	6 (23.1)
Mutations at screening, n (%)		
T315I alone	46 (95.8)	43 (95.6)
T315I and E255K	1 (2.1)	1 (2.2)
T315I and E355G	1 (2.1)	1 (2.2)
*BCR::ABL1*^IS^ at screening, n (%)		
>0.01% to 0.1%	0	0
>0.1% to 1%	8 (16.7)	8 (17.8)
>1% to 10%	11 (22.9)	11 (24.4)
>10%	26 (54.2)	26 (57.8)
Atypical /e1a2/unknown transcripts^b^	3 (6.3)	0

*ECOG* Eastern Cooperative Oncology Group, *TKI* tyrosine kinase inhibitor.

^a^Prior TKIs included dasatinib (n = 5), nilotinib (n = 2), and radotinib (n = 1).

^b^e19a2 transcript, e13a3 (b2a3) transcript, no transcript detected (n = 1, each).

### Efficacy

**Fig. 1:**
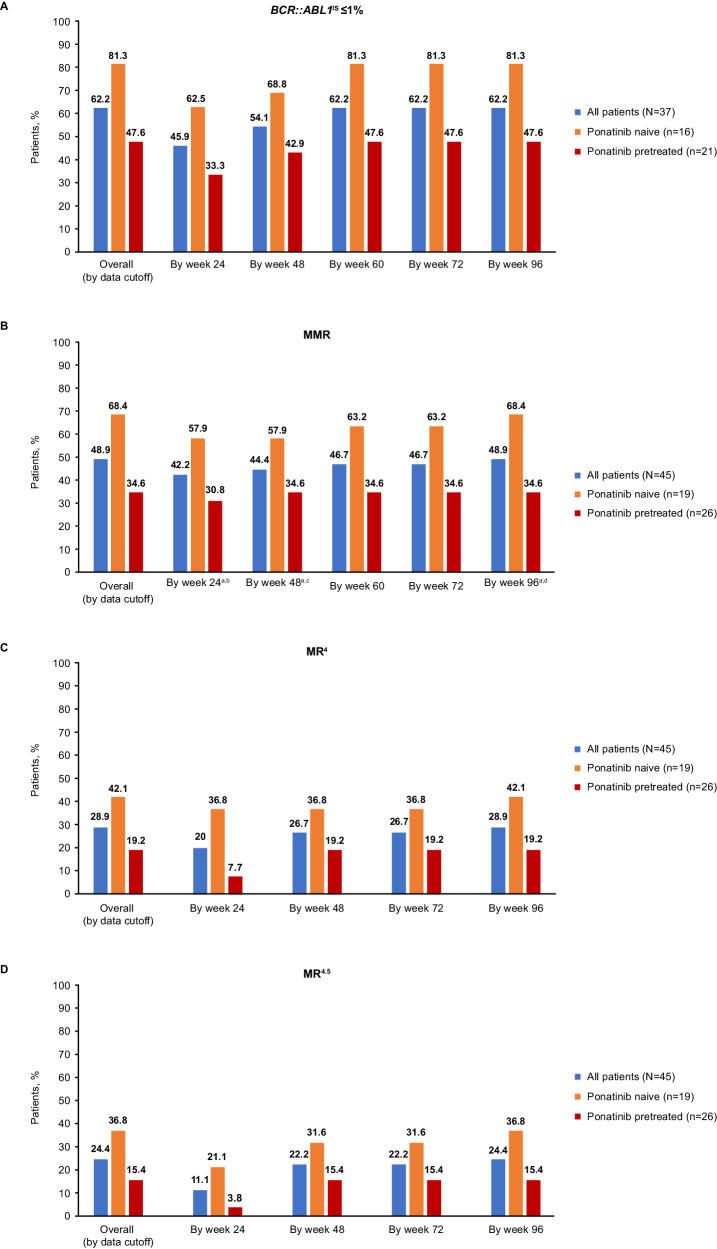
Cumulative molecular response in ponatinib-pretreated and -naive patients without the indicated response at baseline. Cumulative (**A**) *BCR::ABL1*^IS^ ≤1%, (**B**) MMR, (**C**) MR^4^, and (**D**) MR^4.5^ in evaluable patients. IS International Scale, MMR major molecular response, MR^4^ *BCR::ABL1*^IS^ ≤0.01%, MR^4.5^ *BCR::ABL1*^IS^ ≤0.0032%. ^a^ CIs are based on the Clopper–Pearson method. 95% CIs were used for all patients, and 90% CIs were used for ponatinib-pretreated and -naive patients. ^b^ The rate of MMR by week 24 was 42.2% (95% CI, 27.7–57.8%) in all patients, 57.9% (90% CI, 36.8–77.0%) in ponatinib-naive patients, and 30.8% (90% CI, 16.3–48.7%) in ponatinib-pretreated patients. ^c^ The rate of MMR by week 48 was 44.4% (95% CI, 29.6–60.0%) in all patients, 57.9% (90% CI, 36.8–77.0%) in ponatinib-naive patients, and 34.6% (90% CI, 19.4–52.6%) in ponatinib-pretreated patients. ^d^ The rate of MMR by week 96 was 48.9% (95% CI, 33.7–64.2%) in all patients, 68.4% (90% CI, 47.0–85.3%) ponatinib-naive patients, and 34.6% (90% CI, 19.4–52.6%) in ponatinib-pretreated patients.

**Fig. 2:**
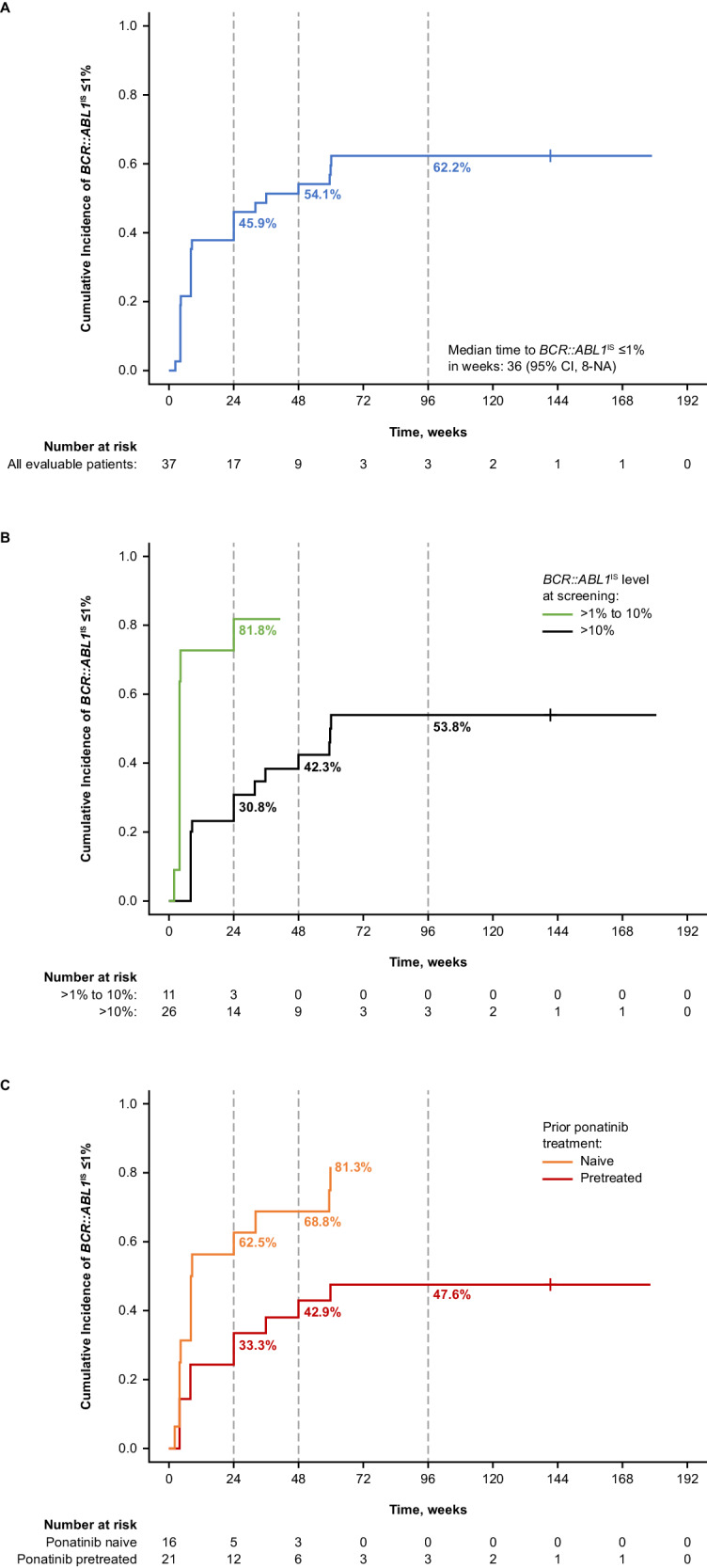
Cumulative *BCR::ABL1*^IS^ ≤1% by time point in patients with baseline *BCR::ABL1*^IS^ > 1%^a^. Cumulative *BCR::ABL1*^IS^ ≤1% in all evaluable patients (**A**) overall, (**B**) by *BCR::ABL1*^IS^ at baseline, and (**C**) by prior treatment with ponatinib. IS International Scale. ^a^ Treatment discontinuations for any reason were treated as competing events.

Of the 48 total patients, 3 were excluded from efficacy analyses due to atypical *BCR::ABL1* transcripts at baseline (n = 2) or no transcripts detected (n = 1) (Table 2; Supplementary Fig. S1). Of the remaining 45 patients, 37 had *BCR::ABL1*^IS^ > 1% at baseline and were evaluable for *BCR::ABL1*^IS^ ≤1% achievement (Fig. 1). Twenty-three (62.2%) patients achieved *BCR::ABL1*^IS^ ≤1% with 17 (45.9%) doing so by week 24 (Fig. 2). The response level of most (6 of 8) patients with *BCR::ABL1*^IS^ ≤1% at baseline deepened by ≥2 logs compared with baseline with treatment; 2 of these 8 patients lost this response.

**Fig. 3:**
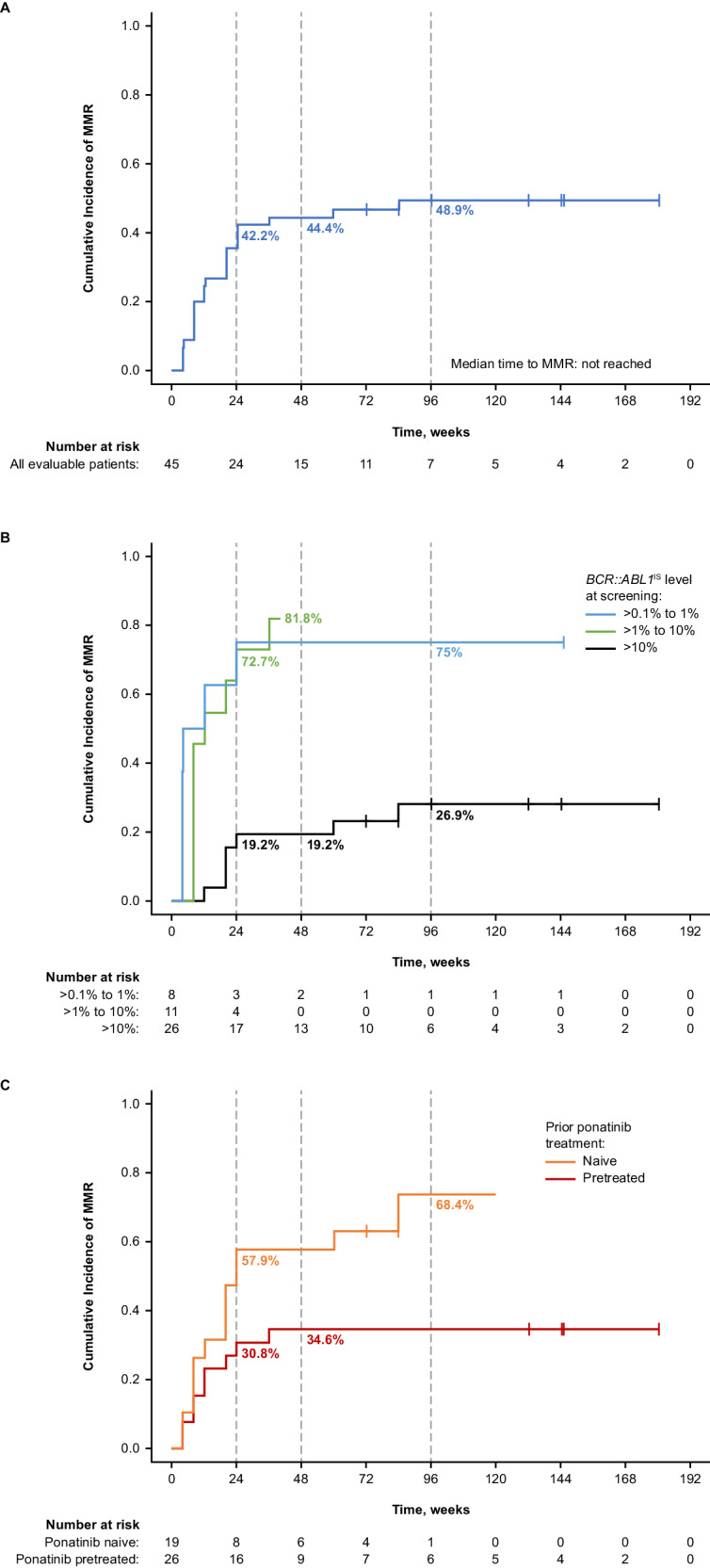
Cumulative MMR by time point in patients not in MMR at baseline. ^a^ Cumulative MMR in all evaluable patients (**A**) overall, (**B**) by *BCR::ABL1*^IS^ at baseline, and (**C**) by prior treatment with ponatinib. IS International Scale, MMR major molecular response. ^a^ Treatment discontinuations for any reason were treated as competing events.

All 45 evaluable patients had *BCR::ABL1*^IS^ > 0.1% at baseline and were evaluable for MMR. Twenty-two (48.9%) patients achieved MMR, with 19 (42.2%) doing so by week 24 (Fig. 3). Two (9.1%) of these 22 patients achieved MMR after dose reduction to 160 mg BID to manage AEs. MMR was achieved in 6 (75%), 9 (81.8%), and 7 (26.9%) patients with *BCR::ABL1*^IS^ >0.1% to ≤1%, >1% to ≤10%, and >10% at baseline (Supplementary Table S4).

Nineteen of 22 patients who achieved MMR maintained MMR by data cutoff. All 3 patients who lost MMR discontinued study treatment (1 due to technical reasons [could not travel due to COVID-19 and was moved to the managed access program] 1 withdrew consent, and 1 to receive transplant). The Kaplan–Meier estimated rate of durable MMR at week 96 was 84% (95% CI, 68.1–100.0%). Deep molecular responses were also observed: 9 (20.0%), 12 (26.7%), and 13 (28.9%) patients achieved at least MR^4^ including 5 (11.1%), 10 (22.2%), and 11 (24.4%) who achieved MR^4.5^ by week 24, 48, and 96, respectively (Fig. 1). Of the 13 patients who achieved at least MR^4^ by week 96, 6 patients had *BCR::ABL1*^IS ^≤1% at baseline and the other 7 patients had *BCR::ABL1*^IS ^>1% at baseline.

An exploratory analysis of evaluable patients stratified by ponatinib pretreatment status suggested that response rates were higher in ponatinib-naive than -pretreated patients. Thirteen of 16 (81.3%) ponatinib-naive and 10 of 21 (47.6%) ponatinib-pretreated patients achieved *BCR::ABL1*^IS^ ≤1%. MMR was achieved by 13 of 19 (68.4%) ponatinib-naive and 9 of 26 (34.6%) ponatinib-pretreated patients (Fig. 1), including patients who discontinued ponatinib due to intolerance (4/7 [57.1%]), resistance (2/13 [15.4%]), or other reasons (3/6 [50%]) (Supplementary Table S5). Two of 3 patients who lost MMR by data cutoff were ponatinib pretreated. The Kaplan–Meier estimated rate of durable MMR at week 96 was 91% (95% CI, 73.9–100.0%) in ponatinib-naive and 78% (95% CI, 50.6–100.0%) in ponatinib-pretreated patients. Of the 9 ponatinib-pretreated patients achieving MMR by cutoff, 7 had *BCR::ABL1*^IS^ ≤10% at baseline and 2 had *BCR::ABL1*^IS^ > 10% at baseline (Supplementary Table S6).

In all 48 patients, the Kaplan–Meier estimated rate of event-free survival at week 96 was 87% (95% CI, 77–97%) (Supplementary Fig. S2A). The median time to event was not reached. Two patients with additional mutations at baseline did not achieve MMR (Supplementary Table S7); one remained on therapy and achieved *BCR::ABL1*^IS^ ≤1% by data cutoff; the other discontinued the study due to physician decision. Six patients acquired additional mutations during the study (M244V, M351T, F359I, A337T, and F359V). The patient who acquired F359V did so after they had achieved MMR and lost it at week 96 (no mutations detectable at that time); at the next assessment, F359V was detected. *BCR::ABL1*^IS^ levels continued to increase until the patient discontinued treatment to receive a transplant. The 5 remaining patients did not achieve MMR; 4 discontinued treatment (3 due to lack of efficacy, 1 due to disease progression), and 1 (with M244V) remained on study drug and achieved *BCR::ABL1*^IS^ ≤1% by data cutoff.

In patients who received asciminib 150 mg BID (n = 5) or 160 mg BID (n = 6), *BCR::ABL1*^IS^ ≤1% and MMR rates did not increase after week 24 (Supplementary Tables S8 and S9). Among evaluable patients, 1 patient with 150 mg BID and 2 with 160 mg BID were in MMR by week 96.

Of 4 patients in AP (Supplementary Table S10), 2 remained on treatment by data cutoff for ≥72 and ≥168 weeks. These 2 patients had *BCR::ABL1*^IS^ >1% to ≤10% at baseline. One achieved MMR by week 48, the other achieved first MR^4.5^ by week 24, and both maintained responses at cutoff. Two patients had *BCR::ABL1*^IS^ >10% at baseline and discontinued treatment, one after treatment for ≥24 weeks without achieving *BCR::ABL1*^IS^ ≤1% and the other after treatment for 23 days without postbaseline assessment.

### Safety

**Fig. 4:**
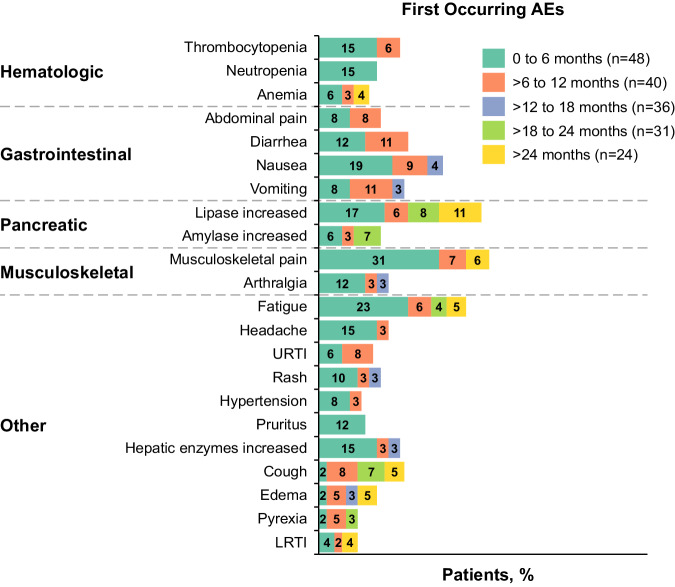
Adverse reactions (≥10% at first occurrence) over time. ^a,b^ADR adverse drug reaction, AE adverse event, LRTI lower respiratory tract infection, URTI upper respiratory tract infection. ^a^ Includes reported AEs and adverse drug reactions. ^b^ Proportions are calculated based on the number of patients at risk of an event (ie, patients ongoing treatment and event-free at the start of the interval). A patient with multiple occurrences of an event in the same time interval is counted only once in that time interval. The safety topics correspond to either single preferred terms or groups of preferred terms according to the adverse drug reaction definitions.

No safety signals were observed with asciminib 150 mg BID or 160 mg BID (Supplementary Table S11), supporting the evaluation of asciminib 200 mg BID. For patients who received asciminib 200 mg BID, all-grade AEs occurring in ≥20% of patients included increased lipase level (29.2%), fatigue (29.2%), nausea (27.1%), and diarrhea (20.8%) (Table 3). Grade ≥3 events occurred in 29 (60.4%) patients; those occurring in ≥10% of patients were increased lipase level (18.8%) and thrombocytopenia (14.6%). Most AEs occurred in the first 6 months of treatment, and incidence rates generally decreased over time (Fig. 4 and Supplementary Fig. S3). AEs led to study discontinuation in 5 (10.4%) patients, including the 2 who died of COVID-19 (Supplementary Table S12); the 3 remaining patients discontinued due to pancytopenia, thrombocytosis, and increased lipase level (n = 1 each). AEs led to dose adjustment or interruption in 19 (39.6%) patients and required additional therapy in 37 (77.1%). No obvious differences in overall safety categories were observed between ponatinib-naive and -pretreated patients; rates of AEs leading to dose adjustment were higher in ponatinib-pretreated (44.8%) than ponatinib-naive (31.6%) patients.

**Table 3:** AEs reported irrespective of study treatment relationship by preferred term reported in ≥10% of patients.

Preferred term, n (%)	All patients N = 48	
	All grades	Grade ≥ 3
No. of patients with ≥1 event	48 (100)	29 (60.4)
Lipase increased	14 (29.2)	9 (18.8)
Fatigue	14 (29.2)	1 (2.1)
Nausea	13 (27.1)	0
Diarrhea	10 (20.8)	1 (2.1)
Vomiting	9 (18.8)	3 (6.3)
Musculoskeletal pain^a^	9 (18.8)	0
Thrombocytopenia	8 (16.7)	7 (14.6)
Headache	8 (16.7)	1 (2.1)
Arthralgia	8 (16.7)	0
Alanine aminotransferase increased	7 (14.6)	3 (6.3)
Abdominal pain	7 (14.6)	3 (6.3)
Cough	7 (14.6)	0
Amylase increased	6 (12.5)	2 (4.2)
Back pain	6 (12.5)	1 (2.1)
Pruritus	6 (12.5)	0
Aspartate aminotransferase increased	6 (12.5)	1 (2.1)
Hypertension	5 (10.4)	3 (6.3)
Anemia	5 (10.4)	3 (6.3)
Edema peripheral	5 (10.4)	2 (4.2)

*AE* adverse event.

^a^Musculoskeletal pain includes preferred terms for musculoskeletal pain and pain in extremity.

Clinically important safety information grouped by special interest categories is reported in Table 4. The most frequently reported categories included gastrointestinal toxicity (47.9%), pancreatic toxicity (31.3%: mainly enzyme elevations; 1 clinical event [grade 2 pancreatitis]), hypersensitivity (27.1%, mainly mild dermatologic events), hepatotoxicity (27.1%, mainly enzyme elevations), and myelosuppression (25%). The most common (≥20%) gastrointestinal toxicities were nausea (27.1%) and diarrhea (20.8%). Grade ≥3 gastrointestinal events occurred in 5 (10.4%) patients. Pancreatic enzyme elevations included increased lipase (31.3%) and amylase (12.5%). Grade ≥3 events occurred in 22.9% of patients (Supplementary Table S13). Events mostly resolved without dose adjustment or with temporary interruption. The grade 2 pancreatitis occurred at day 393 in a patient with prior imatinib, nilotinib, and ponatinib treatment who initiated asciminib 200 mg BID and was receiving asciminib 80 mg BID at onset. Hepatotoxicity included mainly enzyme elevations; grade 3 events occurred in 10.4% of patients. No grade ≥3 hypersensitivity events occurred. Myelosuppression-related any-grade events occurring in ≥10% of patients included thrombocytopenia (18.8%), neutropenia (14.6%), and anemia (10.4%); grade ≥3 events included thrombocytopenia (16.7%), neutropenia (12.5%), anemia (6.3%), and pancytopenia (2.1%).

**Table 4:** Adverse events of special interest.

Safety topic,^a^ n (%)	All patients N = 48	
	All grades	Grade ≥ 3
Gastrointestinal toxicity	23 (47.9)	5 (10.4)
Hypersensitivity^b^	13 (27.1)	0
Hepatotoxicity (including laboratory terms)^c^	13 (27.1)	5 (10.4)
Myelosuppression^d^	12 (25.0)	10 (20.8)
Thrombocytopenia	9 (18.8)	8 (16.7)
Leukopenia	7 (14.6)	6 (12.5)
Erythropenia (anemia)	5 (10.4)	3 (6.3)
Cytopenias affecting >1 lineage	1 (2.1)	1 (2.1)
Pancreatic toxicity (including laboratory terms)	15 (31.3)	11 (22.9)
Pancreatic toxicity (clinical events)	1 (2.1)	0
Edema and fluid retention	8 (16.7)	3 (6.3)
Hemorrhage	9 (18.8)	1 (2.1)
Ischemic heart and CNS conditions	5 (10.4)	2 (4.2)
Ischemic CNS vascular conditions	3 (6.3)	1 (2.1)
Ischemic heart disease	3 (6.3)	1 (2.1)
Arterial occlusive event	4 (8.3)	2 (4.2)
Phototoxicity	1 (2.1)	0
QTc prolongation	1 (2.1)	1 (2.1)

*CNS* central nervous system.

^a^Numbers (n) represent counts of patients. A patient with multiple severity grades for an adverse event is only counted under the maximum grade. MedDRA version 23.1, CTCAE version 4.03, Case Retrieval Strategy version released February 25, 2021.

^b^Mainly mild dermatologic events, including rash, rash maculopapular, dermatitis acneiform, eczema, and urticaria. One event of allergic rhinitis was also observed.

^c^Mainly enzyme elevations.

^d^Myelosuppression includes anemia, leukopenia, thrombocytopenia, and cytopenias affecting >1 lineage.

Four (8.3%) patients had AOEs (Supplementary Table S14); 2 (4.2%) were grade 1 and 2 (4.3%) were grade 3. When adjusted for patient-year exposure, the incidence of all-grade and grade 3 AOEs was 4.3% and 2.2%, respectively (Supplementary Table S15). One patient experienced cerebrovascular accident (grade 3) 13 days after hospitalization due to COVID-19 and died 23 days post-hospitalization due to pneumonia with COVID-19 as a contributing factor. Another patient experienced 3 grade 3 AOEs (peripheral arterial occlusive disease of the right and left side and coronary artery disease), all of which occurred separately over >1300 days of treatment. Percutaneous transluminal angioplasty, electrocardiography, and heart catheterization were performed, and a stent was implanted. Peripheral arterial occlusive disease of both sides later improved to grade 2. Grade 1 AOEs included left carotid artery disease (n = 1) and cerebrovascular accident (n = 1; reported 2 days after the patient discontinued asciminib due to disease progression). Of the 4 patients with AOEs, all had prior exposure to ≥3 TKIs (imatinib [n = 4], nilotinib [n = 3], bosutinib [n = 1], dasatinib [n = 3], and ponatinib [n = 3]). A lower exposure-adjusted incidence of AOEs was observed in ponatinib-naive (2.4%) than in ponatinib-pretreated (5.9%) patients. All patients with AOEs had ≥1 past and/or active CV risk factor at baseline, including arterial hypertension (n = 4), coronary artery disease (n = 1), and cerebrovascular accident (n = 1). No AOEs led to asciminib dose adjustment, interruption, or discontinuation.

Cardiac failure occurred in 1 patient (Supplementary Table S16) who had exposure to 4 prior antileukemic therapies and multiple baseline CV risk factors. On study day 22, pre-existing degenerative aortic valve disease worsened to grade 2, and aortic valve disease (aortic valve vitium) and tricuspid valve incompetence (both grade 1) developed. The patient continued receiving asciminib until hospitalization due to COVID-19 (day 888), a cause of death.

## Discussion

This updated analysis of a phase I trial continues to demonstrate the clinical efficacy and safety of asciminib monotherapy 200 mg BID in patients with T315I-mutated CML-CP—a population that typically has poor prognosis. Molecular responses were observed in few patients who received asciminib 150 mg BID and 160 mg BID doses; the observed efficacy and consistent safety profile in this analysis supports 200 mg BID as the optimal dose for patients with the T315I mutation. After 2 years’ median exposure, most (56.3%) patients continued receiving asciminib. Remarkably, only 4 (8.3%) patients discontinued asciminib due to AEs.

A high proportion (62.2%) of patients achieved *BCR::ABL1*^IS^ ≤1%, most by month 11 (week 48). The achievement of *BCR::ABL1*^IS^ ≤1% or complete cytogenetic response (CcyR) within 12 months predicts long-term survival [1, 19, 20]; achievement at 12 months is associated with higher 6-year OS rate (with CcyR, 93%; without CcyR, 79%) [33] and a lower 5-year rate of disease progression (with CcyR 3%; without major cytogenetic response, 19%) [34]. *BCR::ABL1*^IS^ ≤1% therefore is an important response milestone, particularly for patients in whom ≥2 prior TKIs have not provided MMR [1, 19, 20]. For context, in PACE, 66% and 70% of patients with T315I-mutated CML-CP receiving ponatinib 45 mg QD, the recommended starting dose, achieved CCyR by 12 and 57 months, respectively [13, 18, 35]. In OPTIC, response to treatment was dose dependent, with 51.6%, 35.5%, and 25.3% of patients with T315I-mutated CML-CP at ponatinib starting doses of 45, 30, and 15 mg, respectively, achieving this response by 12 months and 60.0%, 25.0%, and 10.5%, respectively, by 3 years [14, 16].

The achievement of MMR is an important treatment milestone that predicts improved OS, and progression-free survival [1, 33, 36, 37], and is associated with improved durations of CCyR [38]. Almost half of patients (22 [48.9%]), including most patients who achieved *BCR::ABL1*^IS^ ≤1% (16 of 23), achieved MMR. MMR was achieved by patients with all observed baseline *BCR::ABL1*^IS^ levels and in patients who discontinued prior ponatinib for both intolerance and resistance. Responses were sustained, with most patients (19 of 22) who achieved MMR maintaining MMR by data cutoff. In PACE, 56% and 58% of patients with T315I-mutated CML-CP receiving ponatinib 45 mg QD achieved MMR by 12 and 57 months, respectively; 60% of patients who achieved a response by 57 months were estimated to maintain MMR at 5 years [13, 35]. In OPTIC, 34.4%, 24.7%, and 23.1% of patients with CML-CP with or without the T315I mutation at ponatinib starting doses of 45, 30, and 15 mg, respectively, had MMR at cutoff (32 months’ median follow-up) [16].

Deep molecular response (MR^4^ and MR^4.5^) is a strong predictor of OS [39] and can minimize risk of loss of CCyR or MMR [40]. MR^4^ is required for treatment-free remission eligibility [1, 41], and MR^4.5^ has been associated with better event-free survival and failure-free survival than CCyR [39, 42]. In this analysis, >50% of patients who achieved MMR improved to a deeper response level, with 13 (28.9%) and 11 (24.4%) patients achieving MR^4^ and MR^4.5^, respectively.

After 2 years of median exposure, safety results were consistent with the known safety profile of asciminib; no new or worsening safety signals arose in this heavily pretreated patient population. Few patients (5 [10.4%]) experienced AEs leading to discontinuation. Most AEs occurred early (within the first 6 months of treatment); this pattern was also observed in pretreated patients with CML-CP without the T315I mutation from this trial and the phase III ASCEMBL trial [25, 43]. New AEs occurring in ≥10% of patients predominantly after 6 months of treatment included increased lipase level, upper respiratory tract infection, cough, edema, pyrexia, dizziness, increased amylase level, vomiting, anemia, and lower respiratory tract infection; most were grade 1/2.

In the treatment of CML, TKIs have been associated with CV toxicity [18, 29, 44–50], and CV risk profile is a clinical concern in selecting, starting, and monitoring TKI treatment [46]. Results from preclinical models suggest that asciminib may have less potential for cardiotoxicity than ponatinib [51, 52]. In the current analysis, few patients (4 [8.3%]) experienced AOEs. The role of asciminib in these events remains uncertain due to multiple confounding factors, including treatment with multiple prior TKIs and baseline CV risk factors; data from ongoing studies of asciminib in treatment-naive patients will further clarify the CV risk profile of asciminib. In a cohort of 115 patients with CML-CP without the T315I mutation from this trial treated with asciminib doses ranging from 10 to 200 mg QD or BID, with 4.2 years’ median exposure, 10 (8.7%) patients experienced AOEs; none led to treatment discontinuation [25]. In the ASCEND clinical trial of asciminib 40 mg BID starting dose in 101 newly diagnosed patients with median follow-up of 23 months, 1 patient had 2 vascular events [53]. In ASCEMBL, AOEs occurred in 3.2% of patients who received asciminib 40 mg BID with a median follow-up of 1.2 years [24]. In an updated analysis of ASCEMBL with a median follow-up of 2.3 years, the frequency of AOEs rose to 5.1%, but the exposure-adjusted AOE rate (3.0 per 100 patient-years) decreased from that of the primary analysis (3.3) [43, 54]. Patients in these analyses who experienced AOEs while receiving asciminib received several prior TKIs and had multiple baseline CV risk factors [25, 43, 55]. Of 270 patients with CML-CP who received ponatinib 45 mg QD in PACE, 84 (31%) experienced ≥1 AOEs (including CV [16%], cerebrovascular [13%], and peripheral vascular [14%] events) after a median exposure of 32.1 months (range, 0.1–73.0) [13]. OPTIC used ponatinib dose-reduction strategies to assess treatment efficacy while limiting the occurrence of AOEs; after 32 months median follow-up, AOEs were reported in 9.6%, 5.3%, and 3.2% of patients receiving ponatinib 45, 30, and 15 mg, respectively [16]. Ponatinib response-based dose-reduction strategies reduced the risk of AOEs by approximately 60% [21]. In this analysis of X2101, only 1 patient experienced cardiac failure, which occurred contemporaneously with COVID-19 pneumonia from which the patient later died. The role of asciminib remains uncertain due to pretreatment with multiple prior TKIs, baseline CV risk factors, and lack of pretreatment assessment of ejection fraction. While the reported CV events do not constitute a safety signal, risk factors and comorbidities should be closely monitored and managed during asciminib therapy in accordance with clinical practice recommendations.

Pancreatic toxicity poses a safety concern for patients with CML receiving TKI therapy and warrants clinical awareness as enzyme elevations may occur in the absence of evidence of pancreatitis, although the risk factors and mechanism of TKI-associated pancreatic toxicity are unknown [27, 56, 57]. In this analysis with asciminib, pancreatic toxicity events (31.3%) were mainly asymptomatic enzyme elevations. Only 1 patient experienced pancreatitis, which was grade 2 and did not require treatment changes. In a previous analysis of this trial, which included patients with CML-CP/AP with and without the T315I mutation receiving asciminib monotherapy 10 to 200 mg QD or BID (14 months’ median follow-up), clinical pancreatitis occurred in 3% of patients and resolved in all patients within 10 days of discontinuing asciminib [26]. In a cohort of 115 patients with CML-CP without the T315I mutation from this trial, pancreatic enzyme elevations and clinical pancreatitis were reported in 46 (40.0%) and 8 (7.0%) patients, respectively [25]. In contrast, no cases of pancreatitis were reported in ASCEMBL [24, 43]. The asciminib prescribing information recommends monitoring patients monthly or as clinically indicated for pancreatic toxicity, with dose modifications as needed [29].

In the current study, across all observed molecular endpoints, more patients achieved milestones receiving asciminib without prior ponatinib treatment; overall rates were 1.7-, 2-, 2.2-, and 2.4-fold higher in ponatinib-naive than -pretreated patients for *BCR::ABL1*^IS^ ≤1%, MMR, MR^4^, and MR^4.5^, respectively. A similar trend was observed in a real-world analysis of patients with CML treated with asciminib, including 4% with the T315I mutation; in ponatinib-pretreated and -naive patients, respectively, without the indicated response at baseline, 27.3% and 53.3% achieved CCyR, 20% and 52.2% achieved MMR, and 10.5% and 19.4% achieved MR^4.5^ after a median follow-up of 30 months [58]. In the current study, rates of *BCR::ABL1*^IS^ ≤1% and MMR were 2.3- and 3.7-fold higher, respectively, in ponatinib-pretreated patients who discontinued ponatinib due to intolerance versus resistance. Responses were sustained regardless of prior treatment with ponatinib. The safety profile of asciminib was independent of prior ponatinib treatment, with no differences observed between populations in rates of grade ≥3 AEs or AEs requiring additional therapy, reinforcing the safety of asciminib across lines of treatment. AOEs were more frequent in ponatinib-pretreated than -naive patients. The tolerability of asciminib and higher efficacy in ponatinib-naive patients may provide rationale for using asciminib before ponatinib in patients with T315I-mutated CML-CP, although asciminib also retains activity after ponatinib.

In conclusion, asciminib exhibited clinical efficacy, a sustained safety profile, and tolerability in patients with T315I-mutated CML-CP who have limited treatment options. Sustained responses were observed in both ponatinib-naïve and -pretreated patients, and >50% of patients remained on therapy at 2 years’ median exposure. This analysis reinforces the utility of asciminib as a treatment option for patients with T315I-mutated CML-CP, regardless of ponatinib pretreatment status.

### Supplementary information

Supplemental Material

## Data Availability

Novartis is committed to sharing with qualified external researchers, access to patient-level data, and supporting clinical documents from eligible studies. These requests are reviewed and approved by an independent review panel on the basis of scientific merit. All data provided are anonymized to respect the privacy of patients who have participated in the trial in line with applicable laws and regulations. This trial data availability is according to the criteria and process described at www.clinicalstudydatarequest.com. The sequencing data described in this publication will be made available for any qualified request. To submit a request, please contact: novartis.datasharing@novartis.com.
