# Supplementary material for: Asciminib monotherapy in patients with chronic-phase chronic myeloid leukemia with the T315I mutation after ≥1 prior tyrosine kinase inhibitor: 2-year follow-up results
Source: Leukemia. 2024 May 16;38(7):1522–33. doi: 10.1038/s41375-024-02278-8 (PMC11217003; doi:10.1038/s41375-024-02278-8)
Supplement: Supplementary file 1 — Supplemental Material [file 41375_2024_2278_MOESM1_ESM.docx]

**SUPPLEMENTAL**

**METHODS**

**Study Design**

Enrolled patients with chronic myeloid leukemia in chronic phase (CML-CP), accelerated phase (AP), or blast crisis (BC) or Philadelphia chromosome (Ph)+ acute lymphoblastic leukemia (ALL) were allocated into 1 of 5 study arms as previously described: 4 arms assessed patients with CML receiving either asciminib monotherapy or asciminib therapy in combination with another tyrosine kinase inhibitor (imatinib, nilotinib, or dasatinib), and the fifth assessed patients with Ph+ ALL/CML-BC receiving asciminib monotherapy in Ph+ ALL/CML-CP.(25,26) Asciminib was administered with no food for ≥2 hours prior and ≥1 hour after each dose. Each study arm progressed through 3 parts: dose escalation, determination of maximum tolerated dose or the recommended dose for expansion, and dose expansion. The maximum tolerated dose was not reached.(26) In the dose-expansion part of the study, patients were further evaluated for asciminib safety and tolerability and continued asciminib treatment until disease progression, unacceptable toxicity, investigator decision, or withdrawal of patient consent. Patients were not allowed to attempt treatment-free remission on study.

**Patients**

A full description of inclusion and exclusion criteria was reported previously.(1) Key inclusion/exclusion criteria for the T315I cohort included diagnosis of Ph+ CML-CP or -AP and relapsed disease associated with the presence of the T315I mutation after ≥1 TKI, with no other effective therapy available. Patients had Eastern Cooperative Oncology Group (ECOG) performance status of 0–2. Patients were ineligible if they received systematic antineoplastic therapy or any experimental therapy within 14 days or 5 half-lives before the first dose of study treatment, a TKI as a single agent within 5-half-lives, unconjugated monoclonal antibody therapies within 28 days or 5 half-lives, or radiotherapy with either a wide field of radiation within 4 weeks, limited field of radiation for palliation within 1 week, or CNS irradiation for meningeal leukemia. Patients could not have undergone major surgery within 2 weeks before the start of treatment. Patients with a corrected QT interval (QTc) of >480 ms on baseline electrocardiogram or an uncontrolled cardiovascular condition, including ongoing cardiac arrhythmias, congestive heart failure, angina, or myocardial infarction within the past 3 months, were ineligible for study enrollment.

Overall, 79 patients with CML with the T315I mutation (70 in CP and 9 in AP) were enrolled in the study arm in which patients received starting doses of asciminib 20 to 200 mg twice daily (BID) or 80 to 200 mg once daily during the dose-escalation part of the study. Dose increase (up to 250 mg BID) and decrease (down to 5 mg BID) was possible. Forty-eight of these 79 total patients were included in the current analysis of all patients with CML-CP who were assigned to treatment with asciminib 200 mg BID. By the data cutoff (January 6, 2021), all ongoing patients had completed the week 60 visit or discontinued earlier.

**Study Assessments**

Molecular responses were assessed as described previously.(1,2)

**Statistical Analysis**

Event-free survival was estimated using the Kaplan-Meier method; events were defined as treatment discontinuation due to AEs, on-treatment progression to AP/ blast crisis, and on-treatment death for any reason.(1) Adverse events (AEs) were reported as both a number and percentage of patients. A patient with multiple severity grades for an AE was only reported under the maximum grade. Clinically important safety information was identified and presented as previously described.(1)

**References**

1. Mauro MJ, Hughes TP, Kim D-W, Réa D, Cortes JE, Hochhaus A, et al. Asciminib monotherapy in patients with CML-CP without BCR::ABL1 T315I mutations treated with at least two prior TKIs: 4-year phase 1 safety and efficacy results. Leukemia. 2023;37:1048–1059.

2. Hughes TP, Mauro MJ, Cortes JE, Minami H, Rea D, DeAngelo DJ, et al. Asciminib in chronic myeloid leukemia after ABL kinase inhibitor failure. N Engl J Med. 2019;381:2315–2326.

**SUPPLEMENTARY FIGURES**

**Figure S1: Consort Diagram**

**
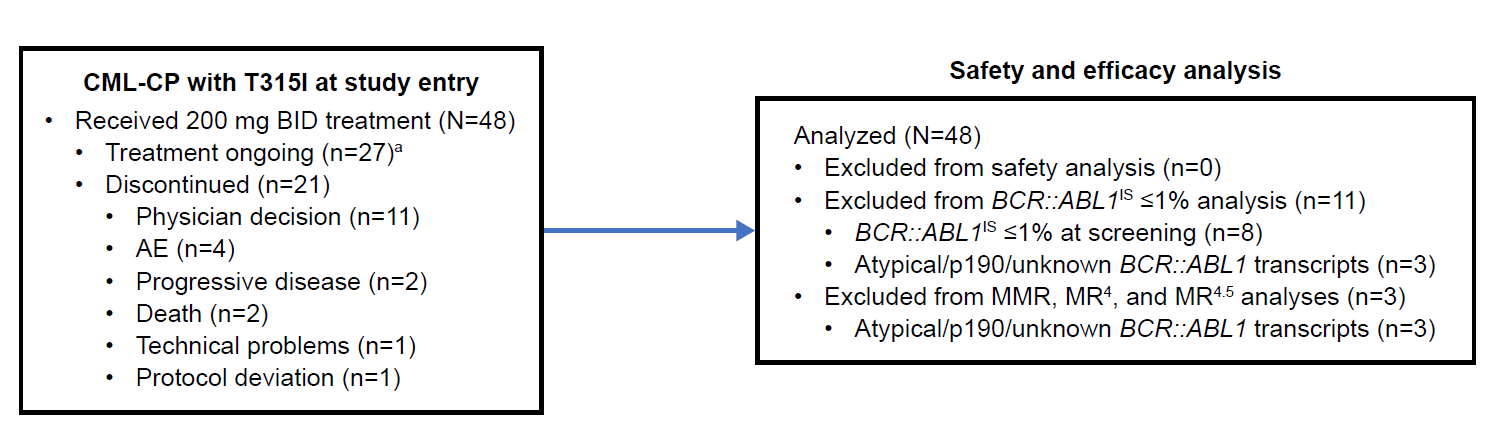
**

AE, adverse event; BID, twice daily; CML-CP, chronic myeloid leukemia in chronic phase; IS, International Scale; MMR, major molecular response; MR^4^, *BCR::ABL1*^IS^ ≤0.01%; MR^4.5^ *BCR::ABL1*^IS^ ≤0.0032%.

^a^ Ongoing at the time of data cutoff: January 6, 2021.

**Figure S2: Event-Free Survival^a^**

**A. Event-Free Survival in Evaluable Patients**

**
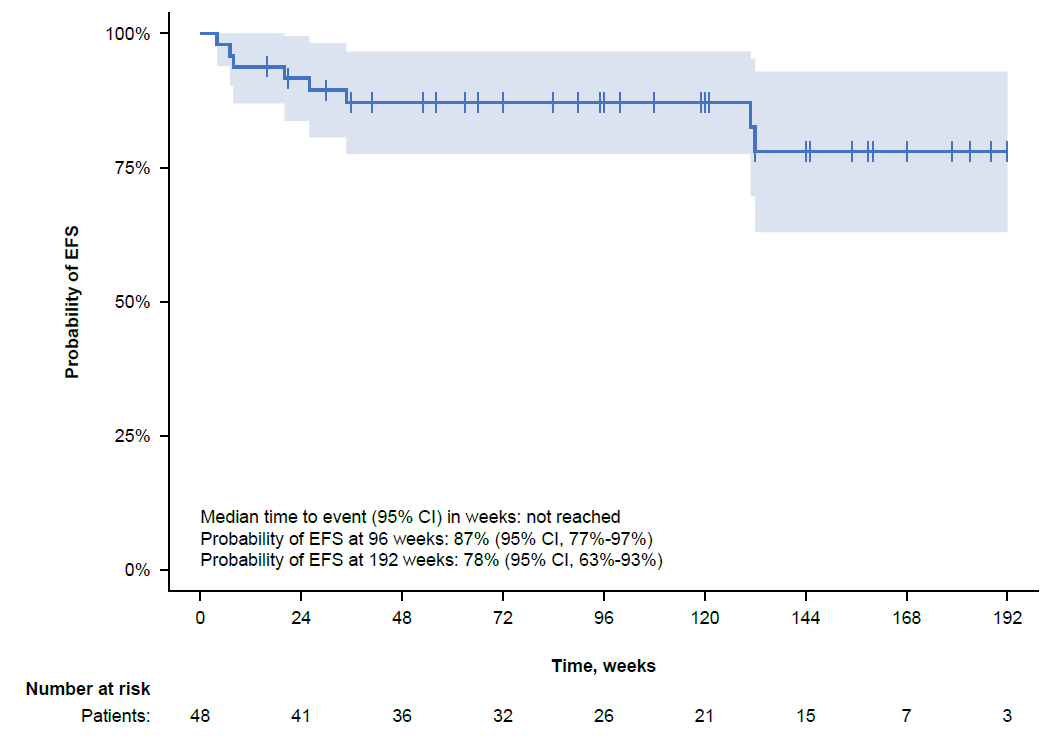
**

**B. Event-Free Survival by *BCR::ABL1*^IS^ ≤1% at Baseline**


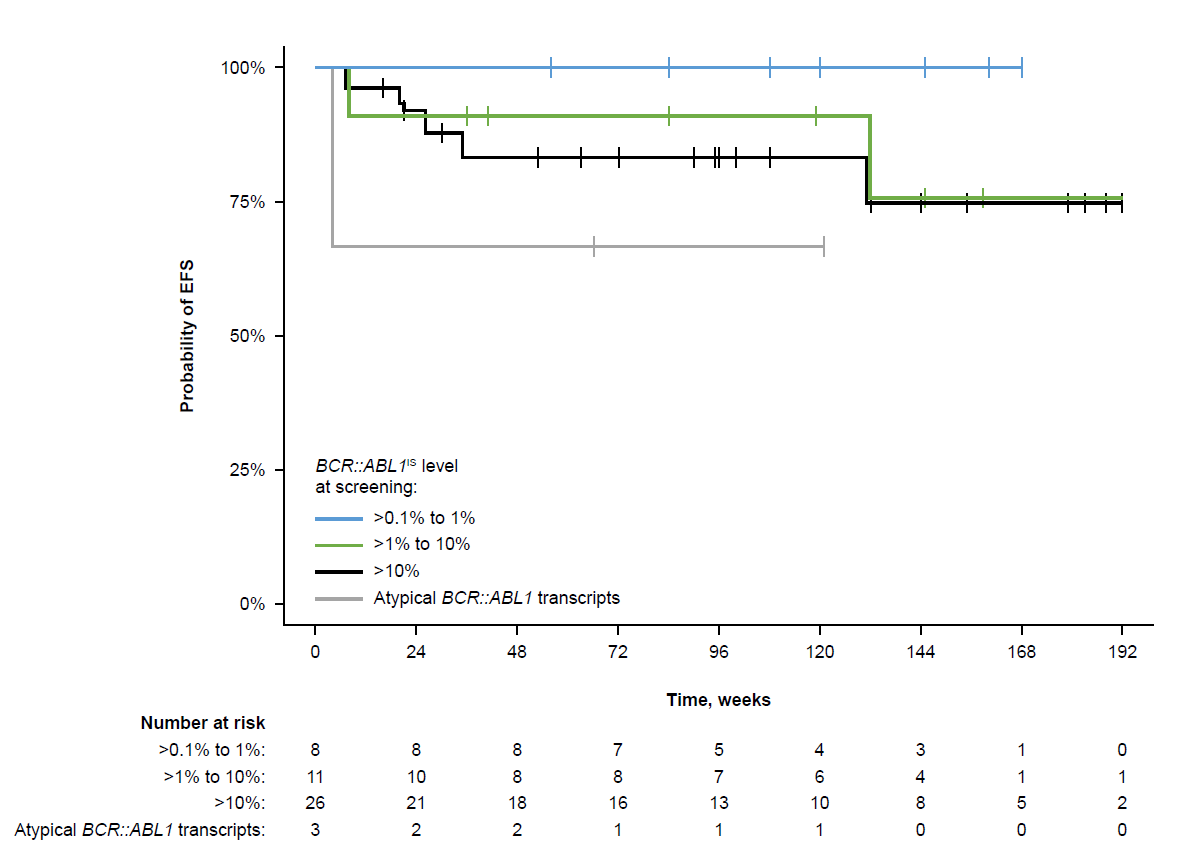


**C. Event-Free Survival by Prior Treatment With Ponatinib**

**
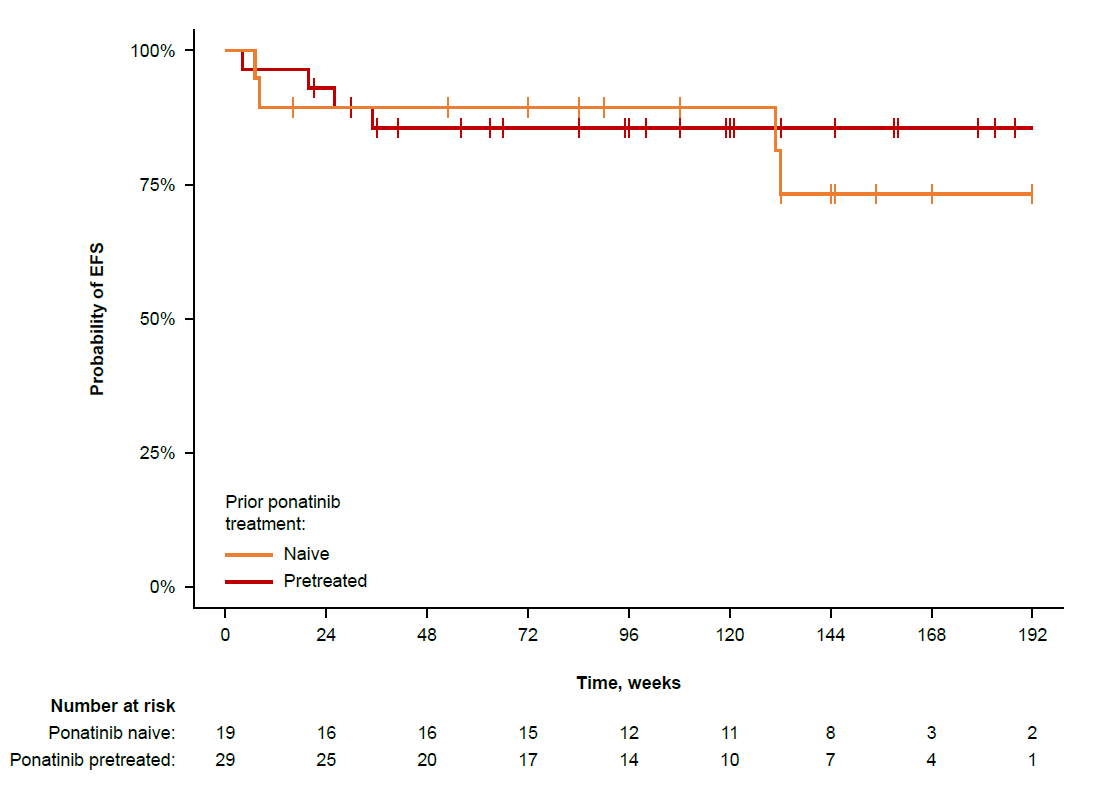
**

EFS, event-free survival; IS, International Scale.

^a^ Event defined as treatment discontinuation due to adverse event, progression to accelerated phase/blast crisis or death due to any reason.

**Figure S3: Adverse Reactions (≥10% at First Occurrence) Over Time^a,b^**


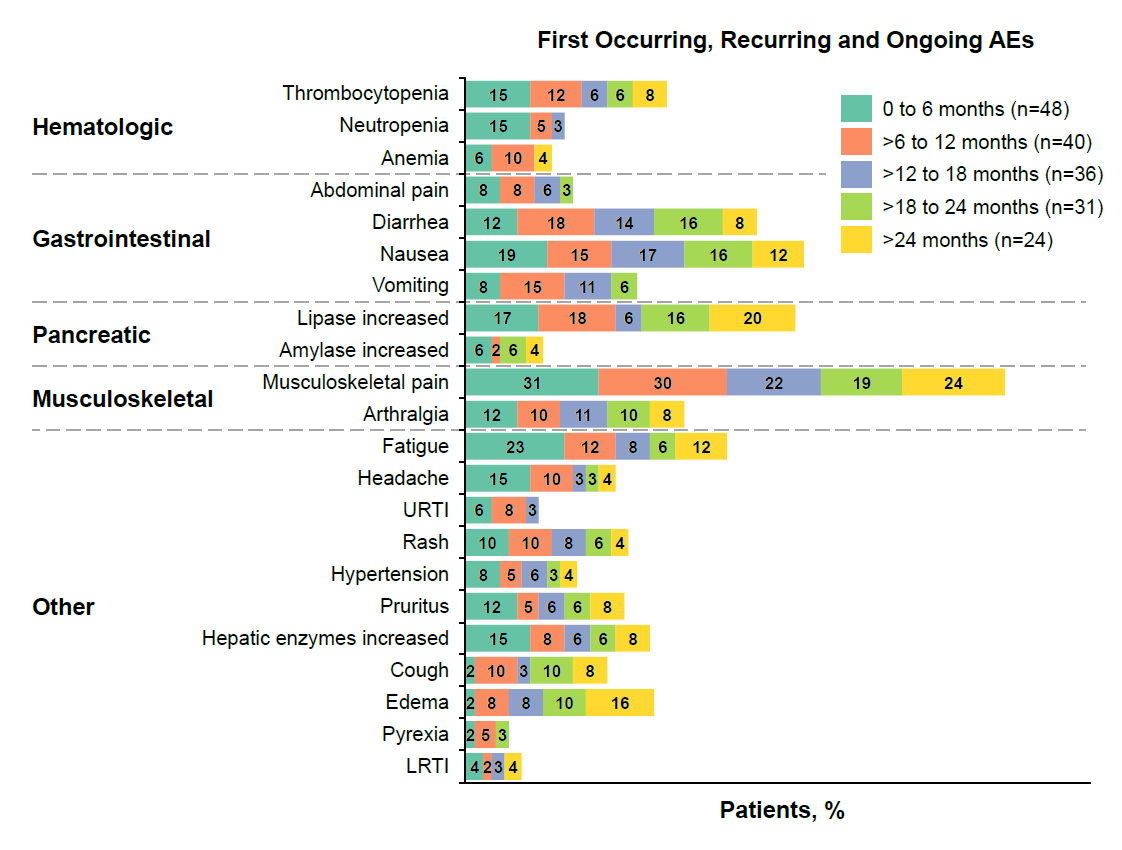


ADR, adverse drug reaction; AE, adverse event; LRTI, lower respiratory tract infection; URTI, upper respiratory tract infection.

^a^ Includes reported AEs and adverse drug reactions.

^b^ Proportions are calculated based on the number of patients at risk of an event (i.e., patients ongoing treatment at the start of the interval). A patient with multiple occurrences of an event in the same time interval is counted only once in that time interval. The safety topics correspond to either single preferred terms or groups of preferred terms according to the adverse drug reaction definitions.

**SUPPLEMENTARY TABLES**

|  | **Responders^a^** | **Nonresponders** | **Evaluable patients** | **All patients** |
| --- | --- | --- | --- | --- |
| **No. of patients on treatment, n (%)** | **n=22** | **n=23** | **N=45** | **N=48** |
| **For** ≥**24 weeks** | 21 (95.5) | 17 (73.9) | 38 (84.4) | 40 (83.3) |
| Receiving 200 mg BID at week 24^b^ | 18 (85.7) | 13 (76.5) | 31 (81.6) | 32 (80.0) |
| **For** ≥**48 weeks** | 21 (95.5) | 13 (56.5) | 34 (75.6) | 36 (75.0) |
| Receiving 200 mg BID at week 48^b^ | 17 (81) | 10 (76.9) | 27 (79.4) | 28 (77.8) |
| **For** ≥**96 weeks** | 18 (81.8) | 8 (34.8) | 26 (57.8) | 27 (56.3) |
| Receiving 200 mg BID at week 96^b^ | 13 (72.2) | 7 (87.5) | 20 (76.9) | 20 (74.1) |

**Table S1: Patients Ongoing Treatment at Full Dose by Time Point**

BID, twice daily.

**^a^** Responders were defined as patients who achieved major molecular response at any time point. Patients not on treatment at a specific time point are those who did not reach the specified visit time point by the cutoff date or discontinued earlier.

^b^ Calculated as a proportion of patients receiving treatment for at least that many weeks.

**Table S2: Patient Demographics and Baseline Clinical Characteristics by Prior Treatment With Ponatinib**

| **Demographic variable** | **Ponatinib pretreated**  **N=29** | **Ponatinib naive**  **N=19** |
| --- | --- | --- |
| **Age, median (range), years** | 60.0 (30-86) | 49.0 (26-77) |
| **Age category, n (%)** |  |  |
| 18 to <65 years | 17 (58.6) | 15 (78.9) |
| ≥65 years | 12 (41.4) | 4 (21.1) |
| ≥75 years | 3 (10.3) | 1 (5.3) |
| **Sex, n (%)** |  |  |
| Male | 22 (75.9) | 15 (78.9) |
| Female | 7 (24.1) | 4 (21.1) |
| **Race, n (%)** |  |  |
| White | 21 (72.4) | 7 (36.8) |
| Asian | 3 (10.3) | 9 (47.4) |
| Unknown | 3 (10.3) | 2 (10.5) |
| Other | 1 (3.4) | 1 (5.3) |
| Black or African American | 1 (3.4) | 0 |
| **Ethnicity, n (%)** |  |  |
| Other | 12 (41.4) | 4 (21.1) |
| Not Reported | 10 (34.5) | 3 (15.8) |
| East Asian | 2 (6.9) | 8 (42.1) |
| Unknown | 2 (6.9) | 2 (10.5) |
| Hispanic or Latino | 2 (6.9) | 1 (5.3) |
| Southeast Asian | 1 (3.4) | 1 (5.3) |
| **ECOG performance status, n (%)** |  |  |
| 0 | 21 (72.4) | 15 (78.9) |
| 1 | 8 (27.6) | 4 (21.1) |
| **No. of prior TKIs** |  |  |
| 1 | 0 | 8 (42.1) |
| 2 | 6 (20.7) | 9 (47.4) |
| 3 | 15 (51.7) | 2 (10.5) |
| ≥4 | 8 (27.6) | 0 |
| **Individual prior TKIs** |  |  |
| Bosutinib | 3 (10.3) | 0 |
| Dasatinib | 19 (65.5) | 14 (73.7) |
| Imatinib | 20 (69.0) | 7 (36.8) |
| Nilotinib | 18 (62.1) | 8 (42.1) |
| Ponatinib | 29 (100) | 0 |
| Radotinib | 1 (3.4) | 3 (15.8) |

ECOG, Eastern Cooperative Oncology Group.

**Table S3: *BCR::ABL1*^IS^ Levels at Screening in Patients Intolerant of and Resistant to Prior Ponatinib Treatment**

| ***BCR::ABL1*^IS^ at screening, n (%)** | **Ponatinib pretreated** | | | | **Ponatinib naive** |
| --- | --- | --- | --- | --- | --- |
|  | **Intolerance** | **Resistance** | **Other** | **Evaluable patients** | **Evaluable patients** |
|  | **n=7** | **n=13** | **n=6** | **n=26** | **n=19** |
| **>0.1% to ≤1%** | 3 (42.9) | 1 (7.7) | 1 (16.7) | 5 (19.2) | 3 (15.8) |
| **>1% to ≤10%** | 1 (14.3) | 3 (23.1) | 2 (33.3) | 6 (23.1) | 5 (26.3) |
| **>10%** | 3 (42.9) | 9 (69.2) | 3 (50.0) | 15 (57.7) | 11 (57.9) |

IS, International Scale.

**Table S4: Cumulative Molecular Response by *BCR::ABL1*^IS^ Levels at Screening**

| **Response category, n (%)** | **>0.1% to ≤1%** | **>1% to ≤10%** | **>10%** | **All patients** |
| --- | --- | --- | --- | --- |
| ***BCR::ABL1*^IS^ ≤1%^a^** | **NA** | **n=11** | **n=26** | **N=37** |
| Overall | NA | 9 (81.8) | 14 (53.8) | 23 (62.2) |
| By week 24 | NA | 9 (81.8) | 8 (30.8) | 17 (45.9) |
| By week 48 | NA | 9 (81.8) | 11 (42.3) | 20 (54.1) |
| By week 72 | NA | 9 (81.8) | 14 (53.8) | 23 (62.2) |
| By week 96 | NA | 9 (81.8) | 14 (53.8) | 23 (62.2) |
| **MMR^b^** | **n=8** | **n=11** | **n=26** | **N=45** |
| Overall | 6 (75) | 9 (81.8) | 7 (26.9) | 22 (48.9) |
| By week 24 | 6 (75) | 8 (72.7) | 5 (19.2) | 19 (42.2) |
| By week 48 | 6 (75) | 9 (81.8) | 5 (19.2) | 20 (44.4) |
| By week 72 | 6 (75) | 9 (81.8) | 6 (23.1) | 21 (46.7) |
| By week 96 | 6 (75) | 9 (81.8) | 7 (26.9) | 22 (48.9) |

IS, International Scale; MMR, major molecular response; NA, not applicable.

^a^ Response in patients without *BCR::ABL1*^IS^ ≤1% at baseline.

^b^ Response in patients not in MMR at baseline.

**Table S5: Cumulative Molecular Response in Patients Without the Indicated Response at Baseline by Reason for Prior Ponatinib Discontinuation**

| **Response category, n (%)** | **Intolerance** | **Resistance** | **Other** | **Evaluable patients** |
| --- | --- | --- | --- | --- |
| ***BCR::ABL1*^IS^ ≤1%** | **n=4** | **n=12** | **n=5** | **n=21** |
| Overall | 3 (75) | 4 (33.3) | 3 (60) | 10 (47.6) |
| By week 24 | 2 (50) | 2 (16.7) | 3 (60) | 7 (33.3) |
| By week 48 | 2 (50) | 4 (33.3) | 3 (60) | 9 (42.9) |
| By week 72 | 3 (75) | 4 (33.3) | 3 (60) | 10 (47.6) |
| By week 96 | 3 (75) | 4 (33.3) | 3 (60) | 10 (47.6) |
| **MMR** | **n=7** | **n=13** | **n=6** | **n=26** |
| Overall | 4 (57.1) | 2 (15.4) | 3 (50) | 9 (34.6) |
| By week 24 | 4 (57.1) | 2 (15.4) | 2 (33.3) | 8 (30.8) |
| By week 48 | 4 (57.1) | 2 (15.4) | 3 (50) | 9 (34.6) |
| By week 72 | 4 (57.1) | 2 (15.4) | 3 (50) | 9 (34.6) |
| By week 96 | 4 (57.1) | 2 (15.4) | 3 (50) | 9 (34.6) |

IS, International Scale; MMR, major molecular response.

**Table S6: Cumulative Molecular Response by Data Cutoff in Evaluable Ponatinib-pretreated Patients Without the Indicated Response at Baseline by *BCR::ABL1*^IS^ Levels at Screening**

| **Response category, n (%)** | **≤10%** | **>10%** | **Evaluable ponatinib-pretreated patients** |  |  |  |
| --- | --- | --- | --- | --- | --- | --- |
|  | **n=11** | **n=15** | **n=26** |  |  |  |
| **MMR** | 7 (63.6) | 2 (13.3) | 9 (34.6) |  | 0 | 2(7.7) |
| **MR^4^** | 4 (36.4) | 1 (6.7) | 5 (19.2) |  |  |  |
| **MR^4.5^** | 3 (27.3) | 1 (6.7) | 4 (15.4) |  |  |  |

IS, International Scale; MMR, major molecular response; MR^4^, *BCR::ABL1*^IS^ ≤0.01%; MR^4.5^ *BCR::ABL1*^IS^ ≤0.0032%.

**Table S7: Baseline and Acquired Mutations**

| **Patients who did not achieve MMR** | **n=23** | **Disposition** | **Additional information** |
| --- | --- | --- | --- |
| **Mutations at baseline^a^** |  |  |  |
| Isolated T315I, n | 21 |  |  |
| Additional mutations, n | 2 |  |  |
| T315I, E255K | 1 | Ongoing | Achieved *BCR::ABL*^IS^ ≤1% |
| T315I, E355G | 1 | Discontinued | Transitioned off study due to PI decision (enrolled in IIT) |
| **Mutations acquired^b^ during the study^a^** |  |  |  |
| None, n | 18 |  |  |
| Mutations, n^c^ | 5 |  |  |
| T315I, M244V | 1 | Ongoing | Achieved *BCR::ABL*^IS^ ≤1% |
| T315I, M351T | 1 | Discontinued | Due to PI decision (lack of efficacy) |
| F359I | 1^d^ | Discontinued | Due to PI decision (lack of efficacy/to receive transplant) |
| T315I, F359I | 1 | Discontinued | Due to PI decision (lack of efficacy) |
| A337T^e^, F359V | 1^d^ | Discontinued | Due to disease progression |
| **Patients who lost MMR** | **n=3** | **Disposition** | **Additional information** |
| **Mutations at baseline^a^** |  |  |  |
| Isolated T315I, n | 3 |  |  |
| Additional mutations, n | 0 |  |  |
| **Mutations acquired during the study^a^, n** |  |  |  |
| None, n | 2 | Discontinued | Withdrew consent (1), technical reasons (patient could not travel to site due to COVID-19 and was moved to a managed access program) (1) |
| Mutations, n^c^ | 1 |  |  |
| F359V^d^ | 1 | Discontinued | Confirmed loss of MMR as of week 96 at which time no mutations including T315I were detected. At next assessment, F359V was detected. *BCR::ABL1* continued to increase until patient discontinued to receive a transplant |

IIT, investigator-initiated trial; IS, International Scale; MMR, major molecular response; PI, primary investigator.

^a^ Mutation status based on Sanger sequencing at central laboratory (measuring mutations between amino acids 35 and 510). Per protocol, mutational analysis was performed when there was unconfirmed loss of response or as needed; when a mutation was detected, additional mutational analyses were to be performed every 3 cycles.

^b^ Acquired mutations defined as those newly detected by Sanger sequencing during treatment. These mutations may have been preexisting, below the detection limit of Sanger sequencing, but detectable by next generation sequencing.

^c^ In patients with the T315I mutation alone at baseline.

^d^ Patient had T315I alone at screening, but follow-up assessment detected only the newly reported mutation(s).

^e^ Myristoyl pocket mutation.

**Table S8: Demographics and Baseline Clinical Characteristic of Patients With CML-CP at 150 mg BID and 160 mg BID Dose Levels**

| **Variable** | **150 mg BID n=5** | **160 mg BID n=6** |
| --- | --- | --- |
| **Age, median (range), years** | 48.0 (25-69) | 58.0 (36-72) |
| **Age category, n (%)** |  |  |
| 18 to <65 years | 3 (60.0) | 4 (66.7) |
| ≥65 years | 2 (40.0) | 2 (33.3) |
| ≥75 years | 0 | 0 |
| **Sex, n (%)** |  |  |
| Male | 4 (80.0) | 4 (66.7) |
| Female | 1 (20.0) | 2 (33.3) |
| **Race, n (%)** |  |  |
| Asian | 5 (100) | 2 (33.3) |
| White | 0 | 3 (50.0) |
| Black or African American | 0 | 1 (16.7) |
| Other | 0 | 0 |
| Unknown | 0 | 0 |
| **Ethnicity, n (%)** |  |  |
| East Asian | 4 (80.0) | 2 (33.3) |
| Southeast Asian | 1 (20.0) | 0 |
| Unknown | 0 | 3 (50.0) |
| Not Reported | 0 | 1 (16.7) |
| Hispanic or Latino | 0 | 0 |
| Other | 0 | 0 |
| **ECOG performance status, n (%)** |  |  |
| 0 | 5 (100) | 5 (83.3) |
| 1 | 0 | 1 (16.7) |
| ≥2 | 0 | 0 |
| Missing | 0 | 0 |
| ***BCR::ABL1*^IS^ at screening, n (%)** |  |  |
| ≤0.0032% | 0 | 0 |
| >0.0032% to 0.01% | 0 | 0 |
| >0.01% to 0.1% | 1 (20.0) | 0 |
| >0.1% to 1% | 0 | 1 (16.7) |
| >1% to 10% | 0 | 2 (33.3) |
| >10% | 3 (60.0) | 2 (33.3) |
| Atypical/e1a2/unknown transcripts* | 1 (20.0) | 1 (16.7) |
| Missing | 0 | 0 |

BID, twice daily; CML, chronic myeloid leukemia; CP, chronic phase; ECOG, Eastern Cooperative Oncology Group; IS, International scale.

**Table S9: Cumulative Molecular Response in Patients With CML-CP With the T315I Mutation at 150 mg BID and 160 mg BID Dose Levels**

|  | **150 mg BID n=5** | **160 mg BID n=6** |
| --- | --- | --- |
| **Evaluable patients, n^a^** | 4 | 5 |
| ***BCR::ABL1*^IS^ ≤1%** |  |  |
| By week 24 | 2 (50.0)^b^ | 3 (60.0)^c^ |
| By week 48 | 2 (50.0)^b^ | 3 (60.0)^c^ |
| By week 72 | 2 (50.0)^b^ | 3 (60.0)^c^ |
| By week 96 | 2 (50.0)^b^ | 3 (60.0)^c^ |
| **MMR** |  |  |
| By week 24 | 1 (25.0)^b^ | 2 (40.0) |
| By week 48 | 1 (25.0)^b^ | 2 (40.0) |
| By week 72 | 1 (25.0)^b^ | 2 (40.0) |
| By week 96 | 1 (25.0)^b^ | 2 (40.0) |
| **MR^4^** |  |  |
| By week 24 | 1 (25.0) | 0 |
| By week 48 | 1 (25.0) | 1 (20.0) |
| By week 72 | 1 (25.0) | 1 (20.0) |
| By week 96 | 1 (25.0) | 1 (20.0) |
| **MR^4.5^** |  |  |
| By week 24 | 1 (25.0) | 0 |
| By week 48 | 1 (25.0) | 1 (20.0) |
| By week 72 | 1 (25.0) | 1 (20.0) |
| By week 96 | 1 (25.0) | 1 (20.0) |

BID, twice daily; CML, chronic myeloid leukemia; CP, chronic phase; IS, International scale; MMR, major molecular response; MR^4^, *BCR::ABL1*^IS^ ≤0.01%; MR^4.5^ *BCR::ABL1*^IS^ ≤0.0032%.

^a^ Corresponds to the number of patients in each treatment group not expressing an atypical/e1a2/unknown transcript and not having a missing evaluation at screening.

^b^ Includes one patient with MMR at baseline.

^c^ Includes 1 patient with *BCR::ABL1*^IS^ ≤1% at baseline.

**Table S10: Demographics and Baseline Clinical Characteristics of Patients With CML-AP**

| **Demographic variable** | **All patients**  **n=4** |
| --- | --- |
| **Age, median (range), years** | 44 (28-78) |
| **Age category, n (%)** |  |
| 18-65 years | 3 (75.0) |
| ≥75 years | 1 (25.0) |
| **Sex, n (%)** |  |
| Male | 2 (50.0) |
| Female | 2 (50.0) |
| **Race, n (%)** |  |
| White | 2 (50.0) |
| Other | 1 (25.0) |
| Unknown | 1 (25.0) |
| **Ethnicity, n (%)** |  |
| Other | 1 (25.0) |
| Not reported | 1 (25.0) |
| Unknown | 2 (50.0) |
| **ECOG performance status, n (%)** |  |
| 0 | 3 (75.0) |
| 1 | 1 (25.0) |
| **No. of prior TKIs** |  |
| 1 | 1 (25.0) |
| 2 | 1 (25.0) |
| 3 | 1 (25.0) |
| ≥4 | 1 (25.0) |
| **Individual prior TKIs** |  |
| Bosutinib | 1 (25.0) |
| Dasatinib | 2 (50.0) |
| Imatinib | 2 (50.0) |
| Nilotinib | 4 (100.0) |
| Ponatinib | 2 (50.0) |
| **Mutations at screening, n (%)^a^** |  |
| T315I alone | 3 (75.0) |
| T315I and F359I | 1 (25.0) |
| ***BCR::ABL1*^IS^ at screening, n (%)** |  |
| >1% to 10% | 2 (50.0) |
| >10% | 2 (50.0) |

AP, accelerated phase; CML, chronic myeloid leukemia; ECOG, Eastern Cooperative Oncology Group; IS, International Scale; TKI, tyrosine kinase inhibitor.

**Table S11: Overview of Adverse Events in Patients With CML-CP Who Received Asciminib 150 mg BID or 160 mg BID**

| **Category, n (%)^a^** | **150 mg BID n=5** | **160 mg BID n=6** |
| --- | --- | --- |
| **Adverse events** | 5 (100) | 6 (100) |
| **Grade ≥3 AEs** | 2 (40.0) | 4 (66.7) |
| Hypophosphatemia | 1 (20.0) | 1 (16.7) |
| Acute coronary syndrome | 1 (20.0) | 0 |
| Neutropenia | 0 | 2 (33.3) |
| ALT increased | 0 | 1 (16.7) |
| AST increased | 0 | 1 (16.7) |
| Thrombocytopenia | 0 | 1 (16.7) |
| Anemia | 0 | 1 (16.7) |
| Neutrophil count decreased | 0 | 1 (16.7) |
| Bilirubin conjugated decreased | 0 | 1 (16.7) |
| Hyperglycemia | 0 | 1 (16.7) |
| Blood bilirubin increased | 0 | 1 (16.7) |
| Activated partial thromboplastin time prolonged | 0 | 1 (16.7) |
| Candida infection | 0 | 1 (16.7) |
| Hypernatremia | 0 | 1 (16.7) |
| Hypoalbuminemia | 0 | 1 (16.7) |
| Hypotension | 0 | 1 (16.7) |
| Hypoxia | 0 | 1 (16.7) |
| Multiple organ dysfunction syndrome | 0 | 1 (16.7) |
| Pneumonia klebsiella | 0 | 1 (16.7) |
| Septic shock | 0 | 1 (16.7) |
| Tetany | 0 | 1 (16.7) |
| Transient ischemia attack | 0 | 1 (16.7) |
| **Grade 5 AEs** | 0 | 0 |
| **AEs leading to discontinuation** | 1 (20.0) | 0 |
| **AEs leading to dose adjustment/interruption** | 1 (20.0) | 2 (33.3) |
| **AEs requiring additional therapy** | 5 (100) | 5 (83.3) |

AE, adverse event; BID, twice daily; CML, chronic myeloid leukemia; CP, chronic phase;

CTCAE, Common Terminology Criteria for Adverse Events.

^a^ Numbers (n) represent counts of patients. A patient with multiple severity grades for an AE is only counted under the maximum grade. MedDRA version 23.1, CTCAE version 4.03.

**Table S12: Overview of Adverse Events**

| **Category, n (%)^a^** | **Ponatinib pretreated**  **n=29** | **Ponatinib naive**  **n=19** | **All patients**  **N=48** |
| --- | --- | --- | --- |
| **Adverse events** | 29 (100) | 19 (100) | 48 (100) |
| **Grade ≥3 AEs** | 18 (62.1) | 11 (57.9) | 29 (60.4) |
| **Grade 5 AEs** | 0 | 2 (10.5) | 2 (4.2) |
| COVID-19 pneumonia^b^ | 0 | 1 (2.1) | 1 (2.1) |
| Pneumonia^c,d^ | 0 | 1 (2.1) | 1 (2.1) |
| **AEs leading to discontinuation** | 1 (3.4) | 4 (21.1) | 5 (10.4) |
| Pancytopenia | 0 | 1 (2.1) | 1 (2.1) |
| Thrombocytosis | 1 (2.1) | 0 | 1 (2.1) |
| COVID-19 pneumonia | 0 | 2 (4.2) | 2 (4.2) |
| Lipase increased | 0 | 1 (2.1) | 1 (2.1) |
| **AEs leading to dose adjustment/ interruption** | 13 (44.8) | 6 (31.6) | 19 (39.6) |
| **AEs requiring additional therapy** | 23 (79.3) | 14 (73.7) | 37 (77.1) |

AE, adverse event; CTCAE, Common Terminology Criteria for Adverse Events.

^a^ Numbers (n) represent counts of patients. A patient with multiple severity grades for an AE is only counted under the maximum grade. MedDRA version 23.1, CTCAE version 4.03.

^b^ Occurred on treatment (within 30 days after last dose of study treatment).

^c^ Occurred post study discontinuation.

^d^ Pneumonia occurred post COVID-19 and considered COVID-19 related.

**Table S13: Clinically Important Safety Information: Pancreatic Enzyme Elevations and Pancreatitis**

| **Preferred term, n (%)** | **All patients**  **N=48** | |
| --- | --- | --- |
|  | **All grades** | **Grade ≥3** |
| **No. of patients with ≥1 event** | 15 (31.3) | 11 (22.9) |
| Lipase increased^a^ | 15 (31.3) | 10 (20.8) |
| Amylase increased | 6 (12.5) | 2 (4.2) |
| Pancreatitis | 1 (2.1) | 0 |

^a^ Includes preferred terms lipase increased and hyperlipasemia.

**Table S14: Characteristics of Patients With Arterial Occlusive Events^a^**

| **Patient** | **Age (years) /sex** | **AOE term (maximum grade)** | **Study day of event occurrence** | **Relevant active medical conditions and medical history contributing to CV risk** | **Prior antineoplastic medications** |
| --- | --- | --- | --- | --- | --- |
| 1 | 68/M | Cerebrovascular accident (grade 3) | 917 | **Past:** thrombocytosis  **Active:** Von Willebrand disease, hypertension, degenerative aortic and mitral valve disease, hypercholesterolemia, hepatitis B, bradycardia, obesity | Imatinib, dasatinib, nilotinib, hydroxycarbamide |
| 2 | 52/M | Cerebrovascular accident (grade 1) | 2 days after last dose | **Active:** diabetes, depression, insomnia, hypertension | Imatinib, dasatinib, nilotinib, ponatinib |
| 3 | 77/M | Carotid artery disease (grade 1) | 590 | **Active:** hypothyroidism, anemia, hypertension, coronary artery disease | Imatinib, nilotinib, ponatinib |
| 4 | 76/M | Coronary artery disease (grade 3) | 1274 | **Past:** Cerebrovascular accident, sleep apnea  **Active:** hypertension | Imatinib, dasatinib, bosutinib, ponatinib |
|  |  | Peripheral arterial occlusive disease (grade 3) | 505 (left side)  602 (right side) |  |  |
|  |  | Peripheral artery occlusion | – |  |  |

AOE, arterial occlusive event; CV, cardiovascular; F, female; M, male.

**^a^** The safety topic of AOEs included the following preferred terms: acute coronary syndrome, angina pectoris, carotid artery disease, carotid artery stenosis, cerebellar infarction, cerebral arteriosclerosis, cerebral ischemia, cerebrovascular accident, coronary artery disease, embolism arterial, ischemic stroke, myocardial infarction, myocardial ischemia, peripheral arterial occlusive disease, peripheral artery occlusion, and transient ischemic attack.

**Table S15: Clinically Important Safety Information: Arterial Occlusive Events**

| **Category, n (%)** | **Ponatinib pretreated n=29** | **Ponatinib naive n=19** | **All patients N=48** |
| --- | --- | --- | --- |
| No. of patients with ≥1 event | 3 (10.3) | 1 (5.3) | 4 (8.3) |
| Cerebrovascular accident | 1 (3.4) | 1 (5.3) | 2 (4.2) |
| Carotid artery disease | 1 (3.4) | 0 | 1 (2.1) |
| Coronary artery disease | 1 (3.4) | 0 | 1 (2.1) |
| Peripheral arterial occlusive disease | 1 (3.4) | 0 | 1 (2.1) |
| Peripheral artery occlusion | 1 (3.4) | 0 | 1 (2.1) |
| No. of patients with ≥1 grade 3 AE | 1 (3.4) | 1 (5.3) | 2 (4.2) |
| Cerebrovascular accident | 0 | 1 (5.3) | 1 (2.1) |
| Coronary artery disease | 1 (3.4) | 0 | 1 (2.1) |
| Peripheral arterial occlusive disease | 1 (3.4) | 0 | 1 (2.1) |
| **EAIR^a^** | | | |
| **Category** | **Ponatinib-pretreated N=29** | **Ponatinib-naive N=19** | **All patients N=48** |
| PTY, years | 50.4 | 42.2 | 92.6 |
| No. of patients with ≥1 event, n (IR per 100 PTY) | 3 (5.9) | 1 (2.4) | 4 (4.3) |
| Cerebrovascular accident | 1 (2.0) | 1 (2.4) | 2 (2.2) |
| Carotid artery disease | 1 (2.0) | 0 | 1 (1.1) |
| Coronary artery disease | 1 (2.0) | 0 | 1 (1.1) |
| Peripheral arterial occlusive disease | 1 (2.0) | 0 | 1 (1.1) |
| Peripheral artery occlusion | 1 (2.0) | 0 | 1 (1.1) |
| No. of patients with ≥1 grade 3 AE, n (IR per 100 PTY) | 1 (2.0) | 1 (2.4) | 2 (2.2) |
| Cerebrovascular accident | 0 | 1 (2.4) | 1 (1.1) |
| Coronary artery disease | 1 (2.0) | 0 | 1 (1.1) |
| Peripheral arterial occlusive disease | 1 (2.0) | 0 | 1 (1.1) |

AE, adverse event; EAIR, exposure-adjusted incidence rate; IR, incidence rate; PTY, patient treatment years.

^a^ EAIR: number of patients with an event divided by the corresponding sum of the exposure duration for all patients, where duration of exposure in PTY is counted up to the first qualifying event (or end of time at risk for patients without event [ie, last treatment plus safety follow-up period]).

**Supplementary Table S16: Clinically Important Safety Information: Cardiac Failure**

| **Preferred term, n (%)** | **All patients**  **N=48** | |
| --- | --- | --- |
|  | **All grades** | **Grade ≥3** |
| **No. of patients with ≥1 event** | 1 (2.1) | 1 (2.1) |
| Congestive hepatopathy | 1 (2.1) | 0 |
| Ejection fraction decreased | 1 (2.1) | 1 (2.1) |
